# Resistance development in KPC-producing *Klebsiella pneumoniae* under ceftazidime/avibactam-meropenem pressure: KPC-2 Ω-loop mutations attenuate avibactam efficacy

**DOI:** 10.1128/spectrum.00356-26

**Published:** 2026-08-03

**Authors:** Fu-Hao Li, Mei Zheng, Gong-Mei Zhong, Xue-Mei He, Meng-Zhen Hu, Xu Kuang, Jing-Jing Wu, Chang-Min Li, Jia-Ning Wang, Xiao-Yu Zhou, Yang Yu

**Affiliations:** 1 Guangdong Laboratory for Lingnan Modern Agriculture, National Risk Assessment Laboratory for Antimicrobial Resistance of Animal Original Bacteria, College of Veterinary Medicine, South China Agricultural University 554665, Guangzhou, China; 2 State Key Laboratory for Animal Disease Control and Prevention, South China Agricultural University 12526, Guangzhou, China; 3 Guangdong Provincial Key Laboratory of Veterinary Pharmaceutics Development and Safety Evaluation, South China Agricultural University 12526, Guangzhou, China; JMI Laboratories, North Liberty, Iowa, USA

**Keywords:** KPC-producing *Klebsiella pneumoniae*, ceftazidime/avibactam, meropenem, collateral sensitivity, KPC, Ω-loop mutations, avibactam resistance

## Abstract

The ceftazidime/avibactam (CAZ-AVI, CZA) combination is a potent agent against KPC-producing *Klebsiella pneumoniae* (KPC-Kp) infections, but resistance readily emerges via KPC mutations that restore carbapenem susceptibility. Previous studies have shown that the CZA-meropenem (MEM) combination exhibits favorable therapeutic efficacy by exploiting this collateral sensitivity. Here, we investigated *in vitro* how this adjunctive combination impacts resistance evolution in KPC-Kp and delineated the underlying molecular mechanisms. Resistance evolution in 26 KPC-Kp isolates was selected by multipassaging under dual CZA-MEM pressure. The underlying resistant mechanisms were systematically dissected by integrating MICs, enzyme kinetics, molecular docking, and molecular dynamics simulations. In contrast to CZA alone, the CZA-MEM combination significantly suppressed resistance development, but five strains still adapted. Under dual-drug selection, mutations arose solely within KPC but were restricted to the Ω loop. Kinetic analysis showed that these mutations drive CZA resistance (R164S variants chiefly by increasing ceftazidime hydrolysis, R164L variants by weakening AVI inhibition, and D179Y by both mechanisms) while simultaneously restoring MEM susceptibility (all variants reduced MEM hydrolysis). Importantly, every mutant elevated the AVI IC_50_, suggesting this is the optimal evolutionary path for KPC-Kp under CZA-MEM pressure. Mechanistic studies reveal that R164L/T243S mutations destabilize the Ω loop to reposition Ser70 at the active site and confer AVI resistance by impairing acylation, destabilizing the enzyme-inhibitor complex, and potentially accelerating deacylation. Our study provides actionable clues for positioning and deploying different β-lactam agents in the treatment of KPC-Kp infections.

**IMPORTANCE:**

The global rise of KPC-producing *Klebsiella pneumoniae* (KPC-Kp) severely limits available therapeutic options, underscoring the urgent need for strategies that curb resistance evolution. The ceftazidime/avibactam-meropenem combination exploits collateral sensitivity to suppress resistance development, yet adaptive mutations still emerge. However, the mechanisms underlying resistance development under dual-drug pressure remain unclear. This study focused on Ω-loop mutations in KPC under dual-drug pressure, revealing that these mutations concurrently enhance avibactam resistance and restore carbapenem susceptibility, although the potential contribution of additional genetic alterations cannot be fully excluded. These findings offer critical insights for optimizing combination therapies and monitoring resistance mechanisms during treatment of KPC-Kp infections.

## INTRODUCTION

The widespread use of antibiotics since their introduction into clinical practice in the 1940s has led to the rapid emergence and spread of antibiotic resistance (1). The emergence of carbapenem-resistant *Klebsiella pneumoniae* has ushered in a major crisis in clinical treatment. The carbapenems have been the drug of last resort for *K. pneumoniae* (2, 3); however, resistance to these agents is rapidly increasing (4). Multiple carbapenemases—including KPC, metallo-β-lactamases (e.g., NDM and VIM), and OXA-48-like enzymes—contribute to this resistance worldwide (5). Among them, KPC poses a particularly serious clinical challenge due to its global dissemination, plasmid-mediated rapid spread, and its ability to hydrolyze nearly all β-lactams, leaving few therapeutic options (6). KPC-producing *Klebsiella pneumoniae* (KPC-Kp) is a serious threat to public health, animal production, and environmental development. The appearance of KPC-Kp is a now major challenge for One Health (7).

Ceftazidime/avibactam (CAZ-AVI, CZA) has been approved for KPC-Kp treatment since 2015 and has achieved good results (8). However, in just a few years, CZA resistance due to KPC mutations has appeared (9–11), and 284 mutations have been identified to date. The majority of these result in CZA resistance (12). Of particular concern are the mutants KPC-33 and KPC-31 that contain a D179Y mutation and represent the most frequent mutation identified during *in vitro* evolution of CZA resistance and in clinical settings. This mutation disrupts the salt bridge between R164 and D179, resulting in a more flexible Ω loop. This conformational change not only accelerates the hydrolysis rate of ceftazidime (CAZ) but also diminishes the inhibitory efficacy of AVI (13, 14).

The increasing prevalence of CZA-resistant KPC mutations has currently limited the use of CZA (15). CZA can foster resistance via KPC mutations and ultimately results in clinical treatment failure (16). Therefore, treatment plans now rely on the use of CZA in combination with other antibiotic classes to extend the lifespan of CZA. In particular, CZA has been used in combination with aztreonam, amikacin, fosfomycin, tigecycline, polymyxin, and carbapenems (17–21). KPC-Kp infections have been demonstrated to be responsive to CZA and carbapenem combinations and have a strong synergistic effect (22). The reason is most likely because the majority of CZA-resistant KPC mutants exhibit collateral susceptibility to carbapenems (23). In our preliminary study, a collateral-sensitive pair of CZA plus meropenem (MEM) combinations yielded excellent results both *in vitro* and in a murine infection model. In addition, CZA-MEM greatly reduces the probability of resistance evolution, although tests have indicated that resistance evolution was still observed in some strains under CZA-MEM pressure (24). Therefore, the potential application of CZA-MEM should be further explored to broaden the therapeutic options against KPC-Kp.

Here, we employed multipassage resistance selection experiments to systematically analyze the resistance evolution trajectory of KPC-Kp under collateral-sensitive pair CZA-MEM pressure. We further identified key mutation sites and characterized their impact on drug susceptibility, enzyme kinetics, and protein structure. The goal of these experiments was to elucidate resistance mechanisms and provide a theoretical basis for clinical treatment of KPC-Kp.

## MATERIALS AND METHODS

### Resistance development of KPC-Kp under the pressure of antibiotics

A total of 26 KPC-Kp strains (25 clinical isolates from hospitals and 1 from a pig farm) were tested in this study. Figure S1 provides the detailed characteristics of these strains, including strain names, antimicrobial susceptibility data, as well as genotypic features—KPC types, sequence types, resistance genes, plasmid replicon types, and virulence genes—determined by whole-genome sequencing (Fig. S1). Multipassage resistance selection was performed to obtain resistance in KPC-Kp in the presence of CZA combined with MEM. Briefly, bacterial mid-log cultures (1.0 × 10^6^ colony-forming units [CFU]/mL) were exposed to twofold increasing concentrations of either CZA alone or CZA in combination with a fixed low concentration of MEM (0.125 mg·L⁻¹) for overnight incubation at 37°C. CAZ levels were varied from 0.125 to 256 mg·L⁻¹, and AVI was fixed at 4 mg·L⁻¹. Subsequently, the bacterial solution in the 1× and 2× MIC wells were mixed and incubated in 4 mL of fresh MH broth for 5 h at 37°C with shaking at 180 rpm, and daily MIC changes for CZA and MEM were recorded as well. The above procedures were repeated daily for 2 weeks or until the CZA MICs remained >256 mg·L⁻¹ for 3 days. In addition, resistance development of tested strains was considered to have failed if the CZA MICs did not increase for five consecutive days or the strain did not survive under the selective pressure, with bacteria either inhibited or killed. Developed isolates were incubated on drug-free medium for three passages with repeated MIC testing to assess the stability of the resistance phenotype.

### Construction of *bla*_KPC_ plasmids and KPC protein purification

The *bla*_KPC-2_ gene was amplified by high-fidelity PCR from the genomic DNA of the parent isolate, and its mutants (*bla*_KPC-2-D179Y_, *bla*_KPC-2-Del167-168_EL_, *bla*_KPC-2-R164L_, *bla*_KPC-2-R164L/T243S_, *bla*_KPC-2-R164S_, and *bla*_KPC-2-R164S/Ins275NKDD_) were amplified from the corresponding evolved isolates using primers pBAD24-KPC-F and pBAD24-KPC-R (Table S1). For construction of pBAD24-*bla*_KPC_ plasmids, the pBAD24 vector was linearized with *Sal*I and assembled with the respective PCR products by homologous recombination. To generate the pBAD24-*bla*_KPC-2-T243S_ plasmid, site-directed mutagenesis was performed on the pBAD24-*bla*_KPC-2_ plasmid using primers KPC-2-T243S-F and KPC-2-T243S-R (Table S1). The recombinant plasmids were transformed into *Escherichia coli* DH5α and selected on Luria-Bertani (LB) agar containing ampicillin (50 μg/mL). These pBAD24-based constructs were used for MIC determination.

For protein expression, the *bla*_KPC_ coding sequence was amplified from the pBAD24-*bla*_KPC_ plasmid using primers KPC-*EcoR*I and KPC-*Sal*I (Table S1), which introduced *EcoR*I and *Sal*I restriction sites. The pET28a vector and the PCR product were double-digested with *EcoR*I and *Sal*I, ligated with T4 DNA ligase, and transformed into *E. coli* BL21(DE3) with kanamycin selection (50 μg/mL). KPC expression was induced with 1 mM IPTG at 16°C for 20 h. After induction, cells were harvested, washed with PBS, resuspended in buffer A (50 mM sodium phosphate, pH 7.4, 300 mM NaCl, 10 mM imidazole, and 8 M urea), and lysed by low-temperature high-pressure homogenization at 1,040 kPa for five to seven cycles. The lysate was centrifuged at 12,000 rpm for 30 min at 4°C, and the supernatant was filtered through a 0.22 µm membrane. Purification was performed using an ÄKTA system with a Ni-NTA column. The column was equilibrated with buffer A; the filtered supernatant was loaded at a low flow rate; and after washing with buffer A, target proteins were eluted with a linear gradient of buffer B (50 mM sodium phosphate, pH 7.4, 300 mM NaCl, 500 mM imidazole, and 8 M urea) from 0% to 60%, with fractions collected based on UV absorbance at 280 nm. The purified proteins were refolded by stepwise dialysis against PBS containing 10% glycerol and decreasing urea concentrations (6, 4, 2, 1, and 0 M urea) and stored in 10% glycerol at −80°C.

### *K*_m_ and *k*_cat_ for ceftazidime and meropenem cleavage

Enzyme assays were performed by incubating purified KPC-2 or variants with varying concentrations of CAZ or MEM in 10 mM phosphate buffer (pH 7.2) at 25°C. Substrate hydrolysis was monitored by measuring the decrease in absorbance at 257 nm for CAZ (Δε = 9,812 M⁻¹ cm⁻¹) and 297 nm for MEM (Δε = 10,176 M⁻¹ cm⁻¹) every 10 s for 10 min using a multimode plate reader, with all measurements corrected to a 1 cm path length. The enzyme concentrations and substrate concentration ranges used for each variant are detailed in Table S2. All measurements were performed in triplicate. Initial velocities were calculated using the corresponding extinction coefficients, and *K*_m_ and *k*_cat_ values were determined by fitting the initial velocity data to the Michaelis-Menten equation *v* = *k*_cat_[*S*] / (*K*_m_ + [*S*]) in GraphPad Prism 8. When the velocity of substrate hydrolysis could not be saturated by measurable concentrations due to a high *K*_m_, *k*_cat_/*K*_m_ was obtained by fitting the equation *v* = (*k*_cat_/*K*_m_)[*E*][*S*].

### Competitive inhibition assays

Apparent inhibition constants (*K*i app) of KPC-2 variants for CAZ and MEM were determined by competitive inhibition assays using nitrocefin as the reporter substrate. Varying concentrations of CAZ or MEM were first mixed with nitrocefin (final concentration 50 µM) in 10 mM phosphate buffer (pH 7.2) at 25°C. The reaction was initiated by the addition of the purified KPC variant, and nitrocefin hydrolysis was monitored at 489 nm every 10 s for 10 min. Initial velocities (*v*₀) were obtained from the linear portion of the progress curves. The reciprocal initial velocity (1/*v*₀) was plotted against the concentration of CAZ or MEM, and linear regression was performed to obtain the slope and *y*-axis intercept. The apparent *K*i app was calculated as the intercept divided by the slope. All measurements were performed in triplicate. The enzyme concentrations and inhibitor concentration ranges used for each variant are provided in Table S3.

### IC_50_ for avibactam

Each KPC-2 variant was incubated with avibactam at concentrations ranging from 0.0001 to 64 µM (fourfold serial dilution) in 10 mM phosphate buffer (pH 7.2) for 10 min at 25°C. The enzyme concentrations were adjusted according to the specific activity of each variant against nitrocefin to ensure initial velocity conditions: 50 nM for KPC-2, T243S, R164S, and R164S/Ins275_NKDD; and 200 nM for R164L, R164L/T243S, D179Y, and Del168-169_EL. After preincubation, nitrocefin (100 µM final concentration) was added, and hydrolysis was monitored at 489 nm every 30 s for 30 min in triplicate. IC_50_ values were calculated by fitting residual activity versus inhibitor concentration to a four-parameter dose-response curve. The apparent inhibition constant (*K*i app) was not further determined in this study.

### Molecular modeling and dynamic simulations

The crystal structure of the wild-type KPC-2 (PDB ID: 2OV5) was obtained from the Protein Data Bank (http://www.rcsb.org). Using 2OV5 as the template, the structural models of the mutants were generated by homology modeling in SWISS-MODEL. Molecular docking studies of avibactam with both KPC-2 and the R164L/T243S mutant were performed using the docking and covalent docking modules within Schrödinger Maestro 2022. The catalytic serine (Ser70) was defined as the reactive residue, and nucleophilic attack of the Ser70 Oγ atom on the β-lactam carbonyl carbon of AVI was modeled to form the covalent acyl-enzyme complex. The reliability of the resulting complex was validated by comparison with the published crystal structure of the KPC-2–AVI covalent adduct (25). The top-ranked poses from the docking results for each system were selected and subjected to molecular dynamics (MD) simulations. All simulations were conducted using Gromacs 4.6 with a duration of 200 ns. Following the simulations, the resulting trajectories were analyzed according to established protocols using VMD 1.9.3, QtGrace, PyMOL, and the Gromacs analysis toolkit.

### Growth ability of strains before and after resistance development

Single colonies of the parent strain and the D8 and D16 strains, at days 8 and 16 of passage, were examined for growth ability. In brief, mid-log cultures of the bacterial suspensions were diluted 1/100 in 10 mL LB broth and cultivated at 37℃ with 180 rpm shaking. Bacterial growth was monitored at OD_600_ at 3-h intervals for the first 24 h and at 6-h intervals for the next 24 h. Three replicates were conducted for each strain, and the 48-h growth curves were analyzed by GraphPad Prism 8.

### *Galleria mellonella* infection assays

Virulence of strains before and after resistance development was assessed using the *Galleria mellonella* infection assay. To determine the challenge dose, groups of *G. mellonella* (*n* = 8) were injected with 10 µL of bacterial suspensions (10³−10⁹ CFU/mL) into the left posterior foot and cultured at 37°C for 72 h. Death was confirmed when the larvae turned black and became immobile. The challenge dose was set at the bacterial concentration yielding ~50% survival. Subsequently, four groups of *G. mellonella* (*n* = 15) were injected with 10 µL of the challenge dose. Groups A, B, and C were inoculated with the parental strain, D8 and D16, respectively, while group D received 10 µL of normal saline as a control. Survival was recorded 72 h post-infection.

## RESULTS

### Multiple mutations under selective pressure

We performed 16-day passages of our test strains, and *bla*_KPC_ mutations were monitored daily. We successfully induced resistance to CZA in 25 KPC-Kp strains (except KP13) that were cultured in the presence of CZA alone. In addition, combined CZA and MEM resulted in failures of resistance evolution in 21 strains but still occurred in 5 KPC-Kp strains (P05, P13, P60, H10, and H29) (Table S4). These five surviving strains exhibited CZA MICs of 256 mg·L⁻¹, representing 32- to 256-fold increases, which remained stable for more than three consecutive days (Table 1). Concurrently, susceptibility to MEM for four of the five strains (except P05) increased to varying degrees during resistance development. Increases of CZA and MEM MICs were both detected against P05 (>32-fold for CZA and ~2-fold for MEM). In addition, collateral susceptibility to aminoglycosides was observed in all resistant strains, and collateral susceptibility to tigecycline was detected in P13 and H29 only. No significant changes in other β-lactam MIC values (cefepime, piperacillin, ceftriaxone, and aztreonam, except H10) were observed during resistance development (Table 1).

**TABLE 1:** MIC of parent strains and their corresponding developed strains under CZA-MEM^*c*^

Strains		MLST	KPC type	MIC (mg·L⁻¹)										
				MEM	CZA	GEN	AMK	KAN	TGC	CFP	PIP	CTR	ATM	CIP
P05	Parent strain	ST16	KPC-2	128	8	>256	>256	>256	2	>256	>256	>256	>256	>256
	P05-D8^*a*^		KPC-2	256	128	>256	>256	>256	2	>256	>256	>256	>256	>256
	P05-D16^*b*^		KPC-2	>256	>256	>256	>256	>256	2	>256	>256	>256	>256	>256
P13	Parent strain	ST437	KPC-2	256	2	32	4	>256	2	>256	>256	>256	>256	>256
	P13-D8		D179Y	16	>256	32	2	>256	2	256	>256	>256	>256	>256
	P13-D16		D179Y	32	>256	16	2	32	0.5	>256	>256	>256	>256	>256
P60	Parent strain	ST101	KPC-2	>256	1	32	16	>256	0.25	>256	>256	>256	>256	>256
	P60-D8		Del168-169_EL	16	128	16	4	>256	0.25	>256	>256	>256	>256	>256
	P60-D16		Del168-169_EL	16	256	16	2	>256	0.25	>256	>256	>256	>256	>256
H10	Parent strain	ST11	KPC-2	>256	4	1	16	128	4	>256	>256	>256	>256	256
	H10-D8		R164L	32	128	0.5	16	128	4	16	>256	128	32	128
	H10-D16		R164L/T243S	64	>256	0.5	8	64	4	128	>256	256	128	128
H29	Parent Strain	ST11	KPC-2	>256	4	0.25	0.5	1	16	>256	>256	>256	>256	256
	H29-D8		R164S	64	>256	<0.13	0.25	0.5	8	>256	>256	256	128	256
	H29-D16		R164S/Ins275_NKDD	32	>256	<0.13	0.25	0.5	4	256	>256	>256	>256	256

^*a*^ D8 represents the strain from 8th-day passaging in resistance development.

^*b*^ D16 represents the strain from 16th-day passaging in resistance development.

^*c*^ AMK, amikacin; ATM, aztreonam; CIP, ciprofloxacin; CFP, cefepime; CTR, ceftriaxone; GEN, gentamicin; KAN, kanamycin; MEM, meropenem; PIP, piperacillin; TGC, tigecycline.

**Fig 1:**
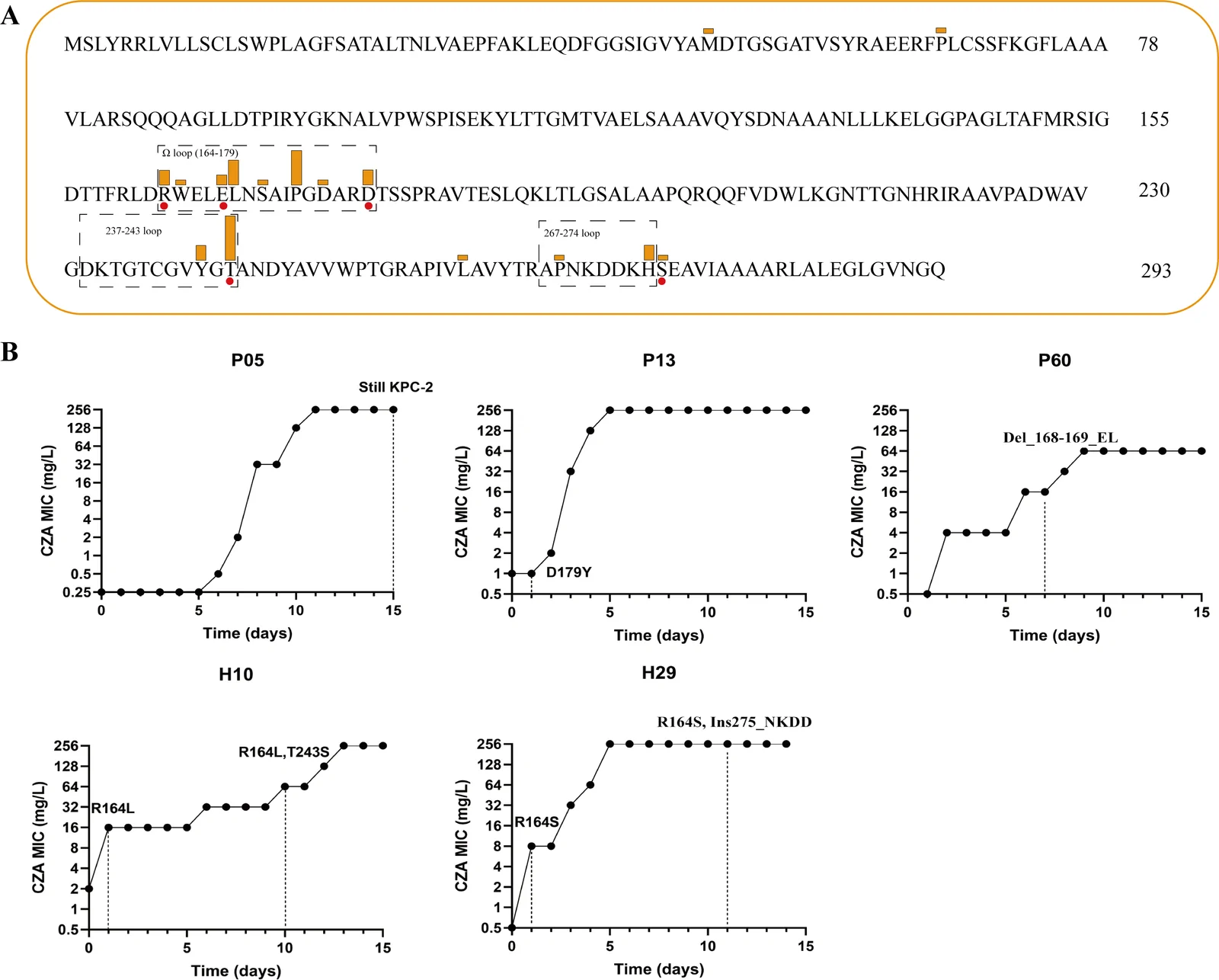
Identification of KPC mutations under CZA and MEM selective pressure. (**A**) Schematic of the KPC-2 primary structure. The dashed boxes highlight three primary mutation regions (amino acids 164–179 [Ω loop], 240–243, and 260–275). The height of the orange bars represents the mutation frequency at each site, and red dots indicate mutations generated under CZA-MEM combination pressure. (**B**) Temporal dynamics and diversity of KPC mutations emerging in five KPC-Kp strains during passage with CZA-MEM.

Mutations induced by CZA alone and CZA-MEM occurred primarily in three regions near the KPC active site (amino acids 164–179 [Ω loop], 240–243, and 260-275) (Fig. 1A). During culture in the presence of CZA alone, a diverse spectrum of mutations emerged within KPC-2/KPC-3 β-lactamases. Specifically, we found recurrent alterations that clustered significantly in the functional critical Ω-loop region, including sites L169P (five isolates), P174L (six isolates), and D179A/N (two isolates). Additional mutations comprised T243A/*P*/G substitutions (eight isolates), along with insertion and deletion events disrupting the Ω-loop structure (Ins171_EV and Del168-169_EL) (Table S4). In contrast, CZA-MEM dual-pressure selected for a markedly restricted mutational repertoire, and KPC mutations were detected in the four isolates, excluding P05, that were also concomitant with elevated CZA MICs (Fig. 1B). Notably, among the diverse β-lactamase genes harbored by the different strains, only *bla*_KPC-2_ acquired mutations under CZA-MEM selective pressure (Table S5). The emergence of KPC mutations was random and differed in four strains, but all the KPC mutations were located in the Ω loop under CZA-MEM selective pressure. Interestingly, R164 and D179 mutations were detected on the first day of CZA-MEM exposure, and sequential mutations occurred twice in H10 and H29. Furthermore, the T243 substitution under CZA-MEM combination therapy differed from the T243A/*P*/G observed under CZA alone, i.e., exclusively T243S (Fig. 1B). However, since whole-genome sequencing was not performed, the potential contribution of mutations in other chromosomal loci cannot be excluded.

### KPC Ω-loop mutations induce seesaw of CZA and MEM

**Fig 2:**
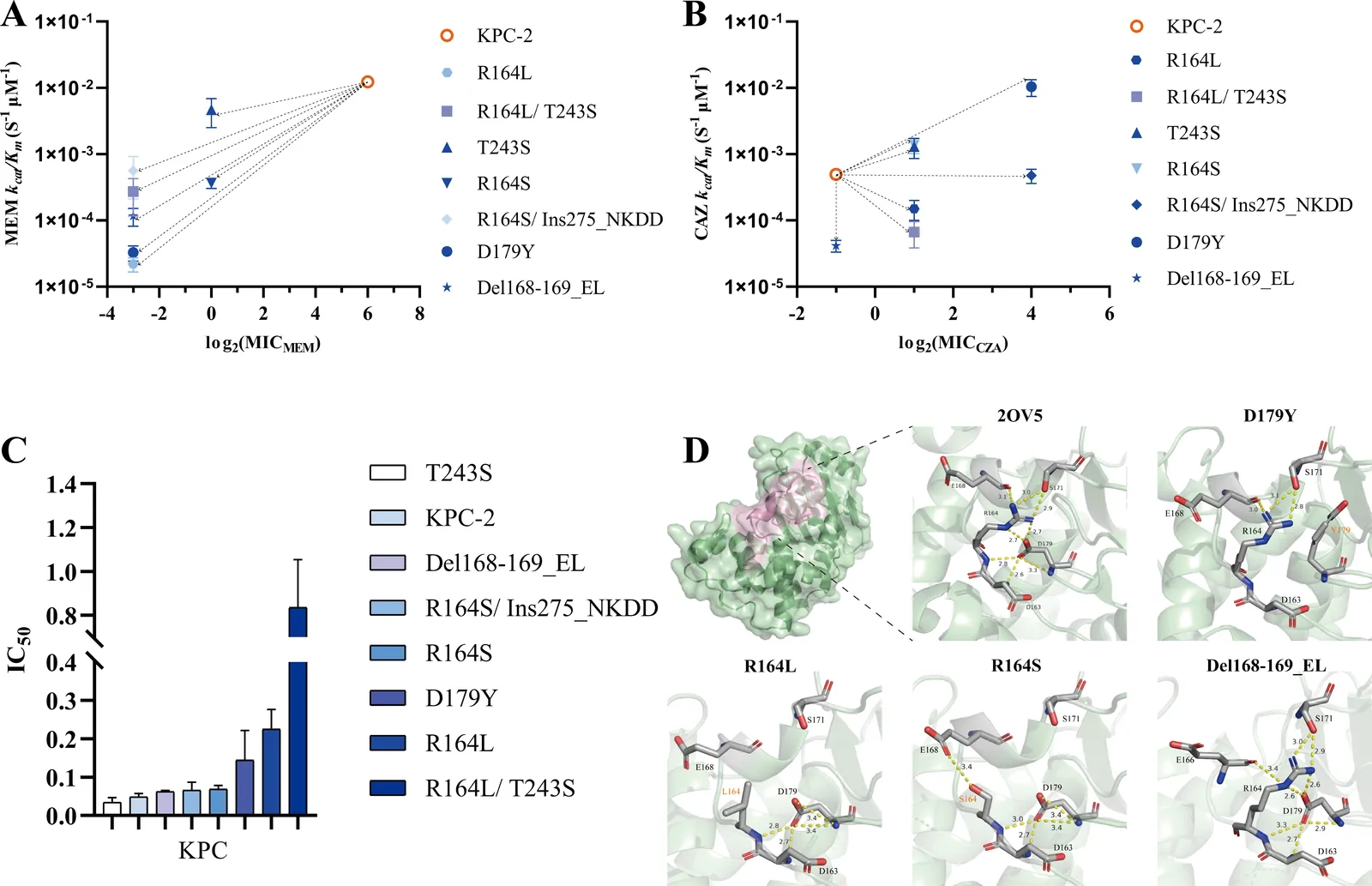
Impact of KPC mutations on enzyme function. Comparison of the catalytic efficiency (*k*_cat_/*K*_m_) of purified KPC-2 and variants toward (**A**) MEM with the corresponding MEM MIC and that toward (**B**) CAZ with the corresponding CZA MIC. (**C**) Half-maximal inhibitory concentration (IC_50_) of AVI against KPC-2 and variant enzymes. (**D**) Structural modeling of the Ω loop, illustrating the hydrogen bonding network (yellow dashed lines) and the impact of specific mutations. Detailed structural analysis is provided in Text S1.

We further investigated the extent that mutations in the KPC Ω loop (selected under CZA-MEM pressure) altered drug susceptibility. For this purpose, the pBAD24 vector harboring *bla*_KPC-2_ with unique KPC mutations was introduced into *E. coli* strain DH5α by electroporation. Successfully constructed strains were analyzed by agarose gel electrophoresis and confirmed using PCR and Sanger sequencing (Fig. S2). The introduction of *bla*_KPC-2_ increased the MIC of CZA from 0.25 to 0.5 and the MIC of MEM from <0.13 to 64 (Table 2). Compared to *bla*_KPC-2_, the introduction of *bla*_KPC-2_ variants increased the MIC for CZA 4- to 32-fold (except Del168-169_EL, which remained at 0.5), whereas the MIC for MEM decreased 64- to >512-fold (Fig. 2A and B). In addition, collateral sensitivities to imipenem, cefepime, ceftriaxone, and aztreonam were observed in these KPC variants (Table 2). The newer β-lactam/β-lactamase inhibitor combinations MEV and IMR retained potent activity against all tested KPC-2 and its variants, with MEV MICs <0.13 mg·L⁻¹ and IMR MICs ranging from 0.5 to 1 mg·L⁻¹ (Table 2).

**TABLE 2:** MICs of *E. coli* DH5α containing different *bla*_KPC-2_ variants^*a*^

Strains	MIC (mg·L⁻¹)									
	CZA	MEM	MEV	IPM	IMR	CFP	CTR	ATM	PIP	
DH5α	0.25	<0.13	<0.13	<0.13	<0.13	<0.13	<0.13	<0.13	1	
DH5α + pBAD24	0.25	<0.13	<0.13	<0.13	<0.13	<0.13	<0.13	<0.13	256	
DH5α + pBAD24-*bla*_KPC-2_	0.5	64	<0.13	128	1	128	>256	>256	>256	
DH5α + pBAD24-*bla*_KPC-2-R164L_	2	<0.13	<0.13	2	1	<0.13	0.5	<0.13	256	
DH5α + pBAD24-*bla*_KPC-2-R164L/T243S_	2	<0.13	<0.13	2	1	32	32	2	64	
DH5α + pBAD24-*bla*_KPC-2-T243S_	2	1	<0.13	64	1	2	32	32	>256	
DH5α + pBAD24-*bla*_KPC-2-R164S_	2	1	<0.13	16	1	2	32	4	256	
DH5α + pBAD24-*bla*_KPC-2-R164S/Ins275_NKDD_	16	<0.13	<0.13	0.5	0.5	2	64	4	256	
DH5α + pBAD24-*bla*_KPC-2-D179Y_	16	<0.13	<0.13	0.5	0.5	<0.13	<0.13	<0.13	128	
DH5α + pBAD24-*bla*_KPC-2-Del168-169_EL_	0.5	<0.13	<0.13	0.5	0.5	2	4	0.06	256	

^*a*^ ATM, aztreonam; CAZ, ceftazidime; CFP, cefepime; CTR, ceftriaxone; CZA, ceftazidime/avibactam (avibactam fixed at 4 mg·L⁻¹); IMR, imipenem/relebactam (relebactam fixed at 4 mg·L⁻¹); IPM, imipenem; MEM, meropenem; MEV, meropenem/vaborbactam (vaborbactam fixed at 8 mg·L⁻¹); PIP, piperacillin.

To obtain biochemical data correlated with the MICs, the pET28a vectors harboring *bla*_KPC-2_ with unique KPC mutations were introduced into *E. coli* BL21(DE3) by electroporation. Successfully constructed strains were verified by agarose gel electrophoresis and confirmed using PCR and Sanger sequencing (Fig. S3). KPC-2 and seven other protein variants were purified by affinity chromatography and analyzed by SDS-PAGE (Fig. S4). Of the seven variants we selected, all except T243S included mutations on the Ω loop. The Ω loop is the most common mutation site and also the region that has the greatest influence on the hydrolytic activity of KPC. Collectively, all variants exhibited a reduction in catalytic efficiency (*k*_cat_/*K*_m_) toward MEM (Table 3), which likely contributes to their restored MEM susceptibility (Fig. 2A). For CAZ, catalytic efficiency was decreased in R164L, R164L/T243S, and Del168-169_EL, whereas it was increased in T243S, R164S, and D179Y, with R164S/Ins275-NKDD showing little change (Table 3; Fig. 2B). The apparent *K*i data further revealed that R164L/T243S, D179Y, and Del168-169_EL exhibited substantially enhanced affinity for MEM, coupled with negligible hydrolysis, suggesting that MEM behaves more as an inhibitor than a substrate for these variants. Conversely, R164L displayed reduced MEM affinity, which may account for its decreased hydrolytic efficiency. In addition, all variants showed enhanced affinity for CAZ relative to KPC-2, with the most pronounced increases observed in R164S/Ins275-NKDD and D179Y (Table 4).

**TABLE 3:** Kinetic parameters of different KPC-2 variants^*a*^

Enzymes	Parameters	Substrates	
		MEM	CAZ
KPC-2	*K*_m_ (μM)	71.88 ± 8.61	>300
	*k*_cat_ (s^−1^)	0.879 ± 0.116	ND
	*k*_cat_/*K*_m_ (μM^−1^·s^−1^)	0.0123 ± 0.0020	0.00050 ± 0.000064
KPC-2 R164L	*K*_m_ (μM)	>512	>300
	*k*_cat_ (s^−1^)	ND	ND
	*k*_cat_/*K*_m_ (μM^−1^·s^−1^)	0.000022 ± 0.0000053	0.00015 ± 0.000050
KPC-2 R164L/T243S	*K*_m_ (μM)	192.93 ± 70.00	>300
	*k*_cat_ (s^−1^)	0.0317 ± 0.0024	ND
	*k*_cat_/*K*_m_ (μM^−1^·s^−1^)	0.00027 ± 0.00016	0.000067 ± 0.000029
KPC-2 T243S	*k*_m_ (μM)	199.07 ± 80.20	>300
	*k*_cat_ (s^−1^)	0.8185 ± 0.1298	ND
	*k*_cat_/*K*_m_ (μM^−1^·s^−1^)	0.0047 ± 0.0022	0.0013 ± 0.00044
KPC-2 R164S	*K*_m_ (μM)	189.90 ± 52.13	>300
	*k*_cat_ (s^−1^)	0.0677 ± 0.0055	ND
	*k*_cat_/*K*_m_ (μM^−1^·s^−1^)	0.00037 ± 0.000064	0.0014 ± 0.00035
KPC-2 R164S/Ins275-NKDD	*K*_m_ (μM)	74.71 ± 44.03	>300
	*k*_cat_ (s^−1^)	0.0322 ± 0.0026	ND
	*k*_cat_/*K*_m_ (μM^−1^·s^−1^)	0.00056 ± 0.00036	0.00048 ± 0.00012
KPC-2 D179Y	*K*_m_ (μM)	>512	1.578 ± 0.4080
	*k*_cat_ (s^−1^)	ND	0.016 ± 0.0024
	*k*_cat_/*K*_m_ (μM^−1^·s^−1^)	0.000033 ± 0.0000084	0.010 ± 0.0029
KPC-2 Del168-169_EL	*K*_m_ (μM)	>512	>300
	*k*_cat_ (s^−1^)	ND	ND
	*k*_cat_/*K*_m_ (μM^**−**1^·s^−1^)	0.00012 ± 0.000035	0.000042 ± 0.0000083

^*a*^ CAZ, ceftazidime; MEM, meropenem. ND, not determined.

**TABLE 4:** Apparent *K*i values for meropenem and ceftazidime against KPC-2 variants^*a*^

Enzymes	MEM (µM)	CAZ (mM)
KPC-2	406.44 ± 69.56	20.76 ± 3.30
KPC-2 R164L	968.80 ± 365.31	7.38 ± 1.51
KPC-2 R164L/T243S	1.02 ± 0.47	8.18 ± 2.88
KPC-2 T243S	–	7.84 ± 3.15
KPC-2 R164S	–	9.00 ± 2.81
KPC-2 R164S/Ins275-NKDD	–	1.79 ± 0.68
KPC-2 D179Y	64.86 ± 6.20	0.99 ± 0.36
KPC-2 Del168-169_EL	1.61 ± 0.29	5.28 ± 2.21

^*a*^ CAZ, ceftazidime; MEM, meropenem. –, no data.

Notably, the IC_50_ to AVI of all six variants containing Ω-loop mutations increased compared to KPC-2 (Fig. 2C). Specifically, R164S, R164S/Ins275_NKDD, and Del168-169_EL variants increased ~1.3-fold, whereas the R164L, R164L/T243S, and D179Y variants increased 4.6-, 17.2-, and 3.0-fold, respectively (Table 5). This explains why R164L and R164L/T243S, despite having reduced hydrolysis efficiency for CAZ, still conferred resistance to CZA. These mutations specifically interfered with the hydrogen bonding network of the Ω loop (Fig. 2D; Text S1).

**TABLE 5:** IC_50_ values of avibactam against different KPC-2 variants

Enzymes	IC_50_ (μM)
KPC-2	0.04860 ± 0.008592
KPC-2 R164L	0.2250 ± 0.05133
KPC-2 R164L/T243S	0.8362 ± 0.2175
KPC-2 T243S	0.03407 ± 0.01256
KPC-2 R164S	0.06912 ± 0.009128
KPC-2 R164S/Ins275-NKDD	0.06562 ± 0.02091
KPC-2 D179Y	0.1442 ± 0.07778
KPC-2 Del168-169_EL	0.06202 ± 0.003328

### KPC Ω-loop mutations mediate the AVI resistance

**Fig 3:**
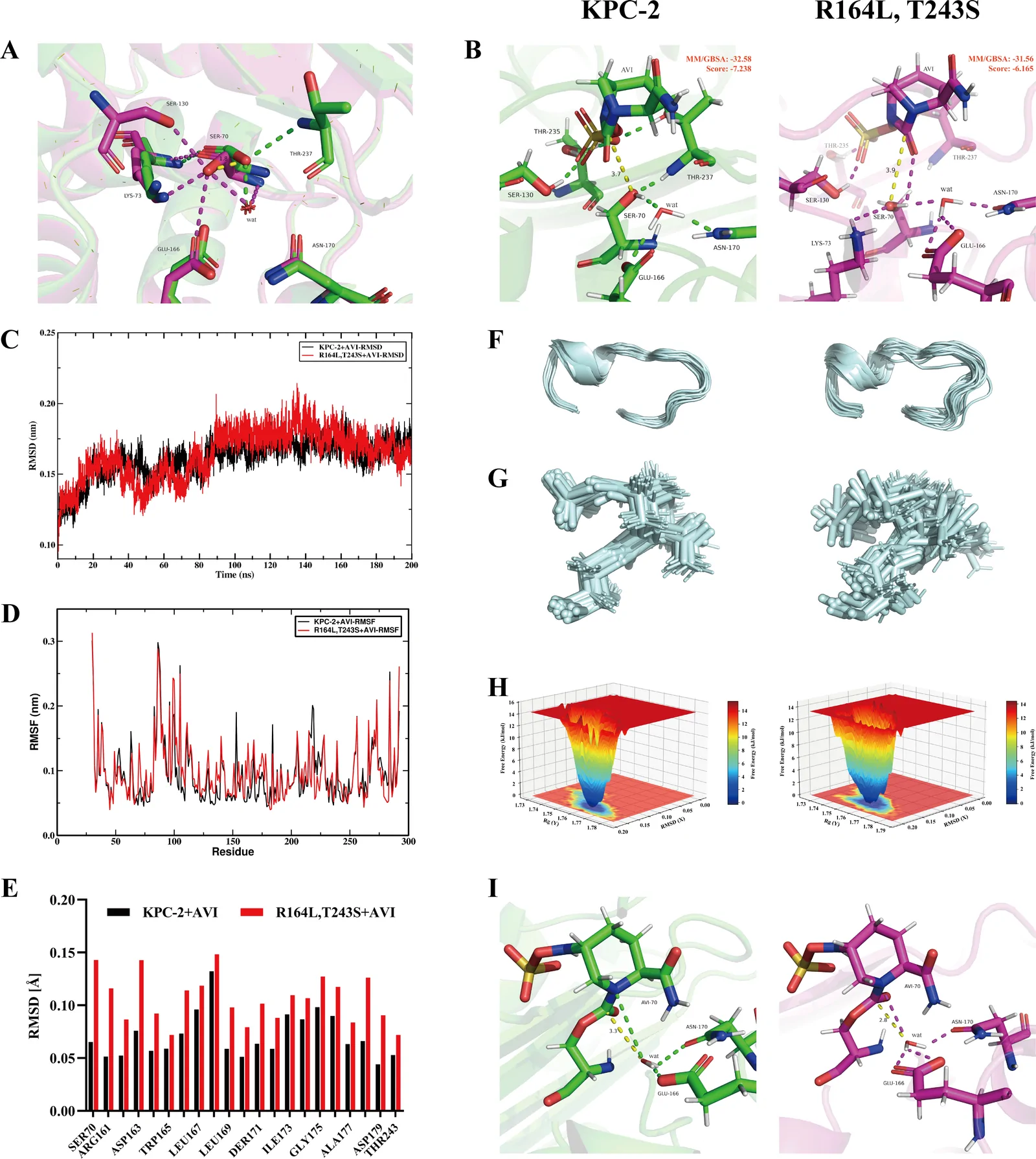
Molecular dynamics (MD) analysis of the AVI resistance mechanism mediated by the KPC-2 R164L/T243S mutations. (**A**) Structural superposition of KPC-2 (green) and the R164L/T243S mutant (purple). (**B**) Predicted binding modes, docking scores, and binding free energies of AVI within the active sites of KPC-2 (left) and the R164L, T243S mutant (right) from molecular docking. (**C**) Root-mean-square deviation (RMSD) and (**D**) root-mean-square fluctuation (RMSF) of the protein backbone during 200 ns MD simulations for the covalent complexes of AVI with KPC-2 (black) and the R164L/T243S mutant (red). (**E**) RMSD of the Ω-loop residues in KPC-2 (black) and the R164L/T243S mutant (red). (**F and G**) Structural superposition of snapshots extracted every 10 ns from the 200 ns MD simulations for the (**F**) Ω loop and (**G**) AVI molecule in KPC-2 (left) and the R164L/T243S mutant (right). (**H**) Free energy landscapes projected against the RMSD (*X*-axis) and the radius of gyration (Rg, *Y*-axis) for the KPC-2 (left) and R164L/T243S mutant (right) complexes with AVI. (**I**) Distance between the deacylating water molecule and the carbonyl carbon of AVI during the 200 ns MD simulations in KPC-2 (left) and the R164L/T243S mutant (right).

From previous studies, it was evident that KPC mutations mediating increased IC_50_ values for AVI played a significant role in the development of CZA and MEM resistance. We therefore selected the R164L/T243S variant that exhibited the most pronounced increase in IC_50_ to investigate potential resistance mechanisms. These mutations significantly altered the hydrogen bond network. Specifically, the salt bridge between R164 and D179 observed in KPC-2 is disrupted in the mutant. In KPC-2, D179 and R164 formed a hydrogen bond network with D163, E168, and S171; however, most of these interactions were lost in the mutant (Fig. 2D). A profound shift in the position of the catalytic residue S70 within the oxyanion hole was observed. In KPC-2, S70 orients toward the oxyanion hole and enables hydrogen bond formation with T237 and a catalytic water molecule, thereby priming the active site for catalysis. In contrast, the hydroxyl group of S70 in the variant was displaced by >1.8 Å and rotated by more than 90° toward N170 and E166. This positional change allowed S70 to form new hydrogen bond interactions with K73 and E166 (Fig. 3A). Subsequent molecular docking of AVI with both KPC-2 and the R164L/T243S indicated that the mutant exhibited increased binding scores and binding free energy compared to KPC-2. Furthermore, the distance between the oxygen atom of S70 and the carbonyl carbon of AVI was increased in the mutant, potentially impeding the acylation process (Fig. 3B).

We then conducted covalent docking of AVI to S70 of KPC-2 and the mutant, followed by 200 ns MD simulations. We applied root-mean-square deviation (RMSD) and root-mean-square fluctuation (RMSF) as key metrics for assessing structural deviation and stability in MD simulations. The RMSD of the KPC-2-AVI complex stabilized at approximately 0.17 nm, while the R164L/T243S-AVI complex stabilized around 0.18 nm (Fig. 3C). The disruption of the hydrogen bond network and salt bridge creates an “open channel” in the middle of the Ω loop, significantly enhancing the flexibility of this region. The residue mobility within the Ω loop was markedly increased in the mutant (average RMSF: ~0.10 Å) compared to KPC-2 (average RMSF: ~0.074 Å) (Fig. 3D through F). Concurrently, the AVI molecule covalently bound to S70 exhibited substantially greater mobility in the mutant (RMSF: 0.143 Å) than in KPC-2 (RMSF: 0.065 Å) (Fig. 3D, E and G). To further investigate the impact of the R164L/T243S mutations on the binding mode of AVI, we compared the free energy landscapes (FEL) of the KPC-2 and mutant complexes. The wild-type system exhibited a single, deep free energy basin that was indicative of a stable and rigid enzyme-inhibitor complex. In stark contrast, the FEL of the mutant system was significantly altered; the deep basin was replaced by a shallower, broader landscape featuring multiple metastable states (Fig. 3H). This result demonstrated that the R164L/T243S mutations substantially reduced the thermodynamic stability of the AVI-bound complex and greatly enhanced its conformational flexibility and heterogeneity. These findings at the MD level revealed a most probable mechanism underlying AVI resistance: the mutations compromised inhibitory efficacy by destabilizing the structural rigidity of the post-binding complex. Furthermore, during the 200 ns simulation, the distance between the deacylating water molecule and the carbonyl carbon of AVI in KPC-2 remained consistently >3.0 Å. In contrast, this distance in the mutant decreased to a minimum of 2.8 Å at specific time points, suggesting a potentially increased rate of deacylation in the mutant (Fig. 3I).

In summary, the R164L/ T243S mutations likely diminished the inhibitory efficacy of AVI by impairing the acylation process, reducing the stability of the enzyme-inhibitor complex and potentially accelerating the deacylation rate.

### Fitness cost

**Fig 4:**
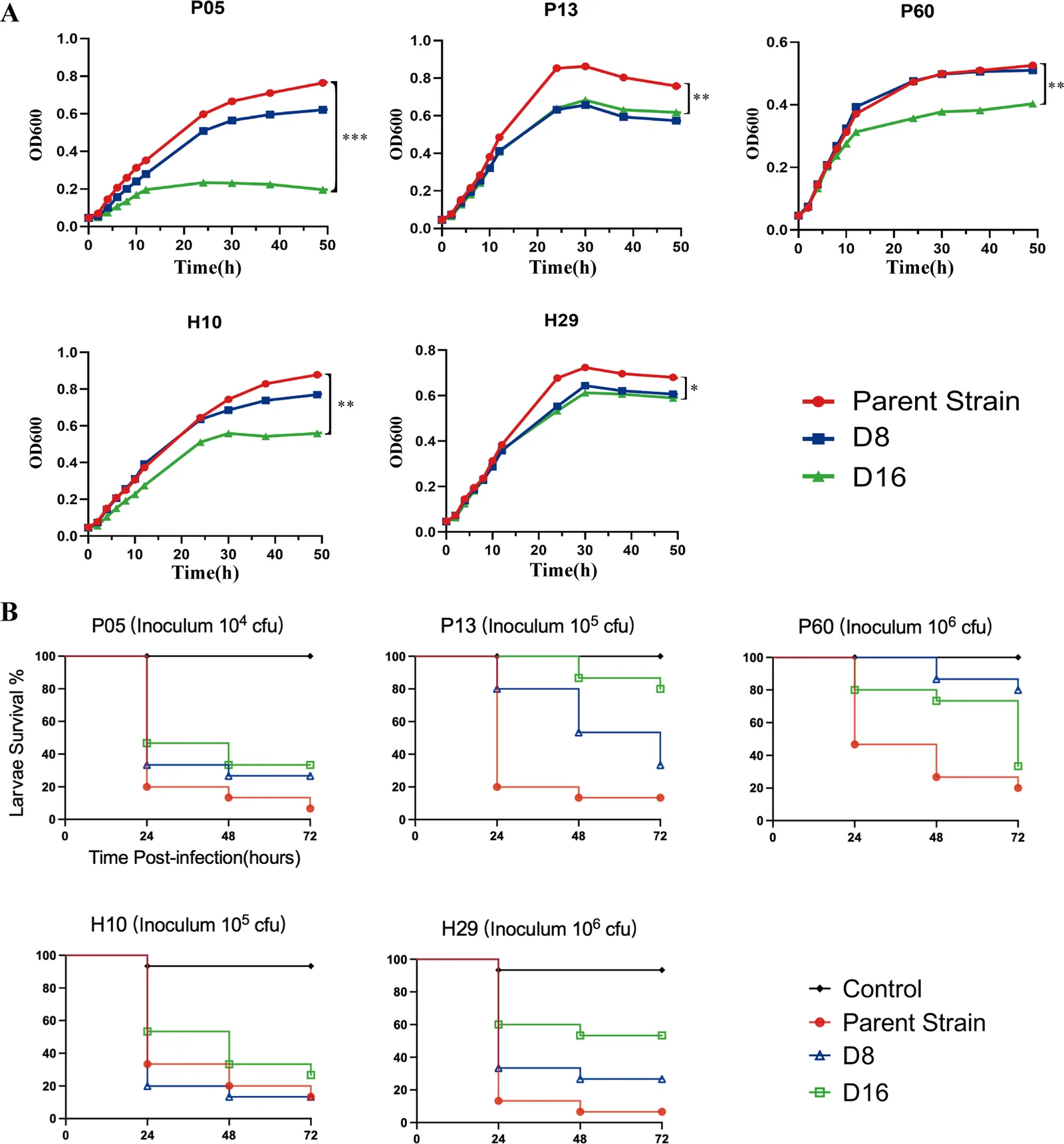
Resistance development in KPC-Kp strains under CZA and MEM combination pressure. (**A**) Growth curves of parent strains and their developed strains (D8, the 8th-day strain from resistance development; D16, the 16th-day strain from resistance development). Significant differences in bacterial growth before and after resistance development are indicated (*P < 0.05, **P < 0.01, ***P < 0.001). (**B**) Virulence assessment using a *Galleria mellonella* infection model. Survival rates of *Galleria mellonella* at 72 h post-infection with parent strains or developed strains (D8 and D16) are shown.

Growth curves of the five strains before and after resistance development revealed a significant fitness cost associated with resistance. In general, compared with the parent strain, the growth ability of the developed strains decreased significantly. For example, the P05 group after 48 h of cultivation possessed OD_600_ values for P05 and P05-D8 of 0.8 and 0.6 while that of P05-D16 was only 0.2. Significant differences in bacterial growth of P05 strains were detected before and after resistance development (*P* < 0.001). Similar results were found in the other four strains as well (*P* < 0.05 or *P* < 0.01) (Fig. 4A).

Virulence was assessed using a *G. mellonella* infection model. The optimal challenge densities for P05, P13, P60, H10, and H29 strains were 10^4^, 10^5^, 10^6^, 10^5^, and 10^6^ CFU/mL per *G. mellonella*, respectively (injection volume: 10 µL). Overall, given the same amount of inoculated bacteria, *G. mellonella* inoculated with the five parental strains were less likely to survive after 72 h compared with those inoculated with the D16 strains. In particular, the P13 group displayed the greatest decrease in virulence, and at 72 h, post-challenge, survival for P13 was 13%, while that of P13-D8 and P13-D16 were 33% and 80%, respectively (Fig. 4B).

## DISCUSSION

Consistent with other studies, mutations induced by CZA alone occurred in several regions around the KPC active site (26–28). However, under the selective pressure of CZA-MEM, a striking convergence was observed; all detected KPC mutations were localized to the Ω loop, a region critical for both substrate hydrolysis and inhibitor binding due to its inclusion of the R164-D179 salt bridge and catalytically important residues like E166 and N170 (13, 29). We propose that the survival of KPC-Kp under dual selective pressure was likely dependent on mutations that reduce AVI-mediated inhibition.

In the midst of the ESBL and carbapenemase rising prevalence lies β-lactamase inhibitors that are the last line of defense that prevent β-lactam antibiotics from becoming useless. AVI is a potent inhibitor of KPC-2 and KPC-3, and it preferentially forms a stable complex with the enzyme to reposition the deacylating water molecule and block hydrolysis, thereby protecting the β-lactam partner (30). The emergence of AVI-resistant KPC variants therefore poses a critical threat to the treatment of KPC-positive organisms. Earlier work has shown that the D179Y substitution abolishes the preferential binding of AVI (14). Our study reveals that R164L mirrors D179Y in several ways, and both disrupt Ω-loop stability (Fig. 3F). Docking analyses revealed higher docking scores and binding energies for the R164L/T243S–AVI complex than for the wild-type, implying a loss of binding priority (Fig. 3B). Moreover, the R164L/T243S–AVI complex was markedly less stable, and the deacylating water was more favorably positioned for hydrolysis (Fig. 3I). Consequently, the IC_50_ for AVI increased 17.2-fold. Although the R164L/T243S mutant hydrolyzed CAZ less efficiently, it remained resistant to the CZA combination (Tables 2, 3 and 5). Greater attention must be paid to KPC mutations that confer AVI resistance to enable the best possible clinical efficacy of treatment.

A pivotal consequence of these Ω-loop mutations was the emergence of collateral sensitivity. Our enzyme kinetics revealed a greatly reduced catalytic efficiency (*k*_cat_/*K*_m_) against MEM in these variants. This is corroborated with studies suggesting that variants like D179Y can cause MEM to act more like a long-term inhibitor than a substrate (14, 31). This collateral susceptibility, also observed against other β-lactams (Table 2), underscores the functional flexibility of the KPC protein; it can evolve specific resistance at the cost of susceptibility to other drugs. This phenomenon highlights the potential for leveraging early mutation monitoring to guide rapid therapeutic adjustments when resistant variants emerge.

However, a potential drawback of CZA-MEM combination therapy should be acknowledged. The addition of MEM, a broad-spectrum carbapenem, may inadvertently exert selective pressure that enriches preexisting MBL-producing *Enterobacterales*, *Acinetobacter baumannii*, and *Pseudomonas aeruginosa* within the host microbiota (32). Since AVI does not inhibit MBLs, the CZA component of the combination would be ineffective against these enriched MBL-producing populations, creating a therapeutic gap (33). Currently, the combination of aztreonam with CZA has emerged as a more reliable strategy for treating MBL-producing organisms, as aztreonam is stable against MBL hydrolysis, and avibactam protects it from serine β-lactamase degradation (34). This issue warrants further clinical investigation to evaluate the ecological impact of CZA-MEM regimens.

Critically, the acquisition of resistance, regardless of the pathway, imposed a significant biological cost. All evolved strains, including those with KPC mutations and the unique P05-D16 strain, exhibited impaired growth (Fig. 4A) and attenuated virulence in the *G. mellonella* model (Fig. 4B). This universal fitness trade-off is a common phenomenon in resistance evolution (35–37) that demonstrates adaptation to the intense pressure of CZA-MEM, which compromises core functions like proliferation and pathogenicity. These may therefore limit the transmission and persistence of these resistant mutants in the absence of antibiotic selection.

In conclusion, our findings demonstrate that supplementing CZA-induced resistance with low-dose MEM can reduce resistance evolution; however, this shifts resistance mutations to the Ω loop and appears to bias toward AVI resistance. Although further investigation is needed, these results provide valuable insights for positioning and optimizing the use of these agents against KPC-Kp infections.

## Data Availability

All sequences have been deposited in the National Center for Biotechnology Information (BioProject number PRJNA510003).

## SUPPLEMENTAL MATERIAL

Text S1, Tables S1 to S5, and Figures S1 to S4. spectrum.00356-26-s0001.docx Biochemical characterization of individual KPC-2 Ω-loop mutations, primer sequences, assay conditions, resistance development data, and β-lactamase mutation screening.

ASM does not own the copyrights to Supplemental Material that may be linked to, or accessed through, an article. The authors have granted ASM a non-exclusive, world-wide license to publish the Supplemental Material files. Please contact the corresponding author directly for reuse.
