# Supplementary material for: Resistance development in KPC-producing Klebsiella pneumoniae under ceftazidime/avibactam-meropenem pressure: KPC-2 Ω-loop mutations attenuate avibactam efficacy
Source: Microbiol Spectr. 2026 Aug 3;14(9):e00356-26. doi: 10.1128/spectrum.00356-26 (PMC13532191; doi:10.1128/spectrum.00356-26)
Supplement: Supplementary file 1 [file spectrum.00356-26-s0001.docx]

**Supplemental files**

**Text S1.** Detailed analysis of biochemical data for each mutation.

D179Y is the most common mutation in the KPC enzyme active site that causes CZA resistance, This alteration results in a breakage of the salt bridge between R164 and D179 of the Ω loop and makes it more flexible (Figure 2D). The mutant exhibited a 21‑fold decrease in *K*i for CAZ, corresponding to a 20‑fold increase in overall catalytic efficiency, consistent with previous studies (1). In addition, IC_50_ for AVI was increased by 3.0× and was lower than the 18.9 × increases previously reported (1). In summary, the MIC of D179Y against CZA was increased 32× (Table 2). Fortunately, the catalytic efficiency of D179Y on MEM was reduced 373× (Table 3). Collectively, these data indicate that meropenem remains a favorable treatment option for infections caused by the D179Y variant.

R164 is located at the opposite end of the salt bridge from D179 and its mutation also affects the stability of the Ω loop. The L164 mutation loses the salt bridge with D179 as well as hydrogen bonding with E168 and S171. S164 is similar to L164 but the side chain hydroxyl group of S164 forms a new hydrogen bond with the carbonyl oxygen of E168 (Figure 2D). The catalytic efficiency of R164L and R164S for MEM decreased by 559× and 33×, respectively. The MIC of R164L and R164S for MEM decreased by >512× and 64×, respectively (Table 2). Although they are all R164 mutations, these mutations that result in CZA resistance are not the same. The catalytic efficiency of R164S for CAZ and IC_50_ for AVI increased by 2.8× and 1.4×, respectively, corresponding to a 4-fold increase in MIC. However, the catalytic efficiency of R164L for CAZ was reduced 3.3×, but the IC_50_ for AVI increased 4.6× that corresponded to a 4-fold increase in the MIC for CZA (Table 2, 3, 5). R164S increases the catalytic efficiency of CAZ, while R164L reduces the inhibitory effect of AVI. The difference is most likely related to the excess hydrogen bond of R164S (Figure 2D).

R164L/ T243S includes both R164L and T243S mutations. The T243S variant alone showed a 2.6-fold reduction in catalytic efficiency for MEM and a 2.6-fold increase in catalytic efficiency for CAZ. A non-significant decrease in IC_50_ for AVI was also noted and was consistent with 4-fold increase in the CZA MIC (Table 2). In addition, the IC_50_ of AVI to R164L and R164L/ T243S increased by 4.6× and 17.2× and this explains why that even though the hydrolysis of CAZ decreased, the MIC of CZA still increased 4× (Table 2, 3, 5). Notably, the *K*i values for MEM in R164L and R164L/ T243S showed opposite trends compared with KPC-2 (Table 4), and the underlying mechanism warrants further investigation.

R164S increased the catalytic efficiency toward CAZ and the IC₅₀ for AVI, but reduced the catalytic efficiency toward MEM, consistent with the MIC changes shown in Table 2. In contrast, the hydrolytic efficiency of R164S/ Ins275_NKDD toward CAZ remained largely unchanged compared with KPC‑2, yet the CZA MIC increased 32‑fold. This is likely because the Ins275_NKDD insertion enables the KPC active site to better accommodate CAZ, which bears a bulky aminothiazole side chain, as supported by the 11.6‑fold decrease in *K*i for CAZ in this variant (Table 4). It is plausible that when R164S/Ins275_NKDD encounters both CAZ and AVI, it preferentially binds CAZ over AVI; however, this hypothesis requires further investigation.

Del168-169_EL is missing one of the two repeating glutamic acid - lysine repeats in the Ω loop (164 -RWELELNSAIPGDARD - 179). Homologous modeling of the protein indicated that the remaining E166 appeared to replace the function of E168 and formed a hydrogen bond network similar to KPC-2 (2OV5), but also disrupted the catalytic triad E166, N170 and K73 (Figure 2D). This was consistent with the observed reduction in *k*_cat_/*K*_m_ for MEM and CAZ. In addition, this was the only variant that both reduced the catalytic efficiency of CAZ and did not significantly raise the IC_50_ against AVI and as such, it maintained the same MIC as KPC-2 against CZA (Table2, 3, 5).

**Table S1.** Primers used for construction of *bla*_KPC_ plasmids and protein expression.

| Primer name | Primer sequence (5’-3’) |
| --- | --- |
| pBAD24-KPC-F | CCATGGTACCCGGGGATCCTCTAGATTCAGTTGGTGTTGACACCG |
| pBAD24-KPC-R | TGCATGCCTGCAGGTCGACTCTAGACCATCGTCAGTGCTCTACAG |
| KPC-2-T243S-F | TGGCTCGGCAAATGACTATG |
| KPC-2-T243S-R | TTTGCCGAGCCATACACTC |
| KPC-*EcoR*I | ATCGCGAATTCCTGACCAACCTCGTCGC |
| KPC-*Sal*I | ATCGCGTCGACTTACTGCCCGTTGACGC |

**Table S2.** Enzyme concentrations and substrate concentration ranges used for kinetic assays of KPC‑2 variants.

| **Enzyme** | **Substrate** | **Enzyme concentrations (nM)** | **Substrate concentration range (µM)** |
| --- | --- | --- | --- |
| KPC-2 | MEM | 50 | 0, 8, 16, 32, 64, 128, 256, 512 |
|  | CAZ | 800 | 0, 60, 80, 100, 120, 150, 200, 300 |
| R164L | MEM | 800 | 0, 8, 16, 32, 64, 128, 256, 512 |
|  | CAZ | 800 | 0, 30, 60, 90, 120, 150, 200, 300 |
| R164L/ T243S | MEM | 800 | 0, 8, 16, 32, 64, 128, 256, 512 |
|  | CAZ | 800 | 0, 30, 60, 90, 120, 150, 200, 300 |
| T243S | MEM | 200 | 0, 8, 16, 32, 64, 128, 256, 512 |
|  | CAZ | 200 | 0, 30, 60, 90, 120, 150, 200, 300 |
| R164S | MEM | 200 | 0, 8, 16, 32, 64, 128, 256, 512 |
|  | CAZ | 200 | 0, 30, 60, 90, 120, 150, 200, 300 |
| R164S/ Ins275_NKDD | MEM | 200 | 0, 8, 16, 32, 64, 128, 256, 512 |
|  | CAZ | 200 | 0, 30, 60, 90, 120, 150, 200, 300 |
| D179Y | MEM | 800 | 0, 8, 16, 32, 64, 128, 256, 512 |
|  | CAZ | 800 | 0, 0.25, 0.5, 1, 2, 4, 8, 16 |
| Del68-169_EL | MEM | 200 | 0, 8, 16, 32, 64, 128, 256, 512 |
|  | CAZ | 800 | 0, 30, 60, 90, 120, 150, 200, 300 |

Abbreviation: CAZ, ceftazidime; MEM, meropenem.

**Table S3.** Enzyme and inhibitor concentrations used for competitive inhibition assays.

| **Enzyme** | **Inhibitor** | **Enzyme concentrations (nM)** | **Inhibitor concentration range (mM)** |
| --- | --- | --- | --- |
| KPC-2 | MEM | 50 | 0.5 0.25 0.125 0.0625 0.031 0.015 0.0078 0 |
|  | CAZ | 10 | 162 145 127 108 90 72 18 0 |
| R164L | MEM | 500 | 5 2.5 1.25 0.625 0.31 0.15 0.078 0 |
|  | CAZ | 500 | 10 5 2.5 1.25 0.625 0.3125 0.15625 0 |
| R164L/ T243S | MEM | 50 | 0.025 0.0125 0.00625 0.0031 0.0015 0.00078 0.00039 0 |
|  | CAZ | 500 | 10 5 2.5 1.25 0.625 0.3125 0.15625 0 |
| T243S | MEM | - | - |
|  | CAZ | 10 | 162 145 127 108 90 72 18 0 |
| R164S | MEM | - | - |
|  | CAZ | 10 | 162 81 40.5 20.25 10.125 5.0625 2.53125 0 |
| R164S/ Ins275_NKDD | MEM | - | - |
|  | CAZ | 50 | 10 5 2.5 1.25 0.625 0.3125 0.15625 0 |
| D179Y | MEM | 500 | 0.5 0.25 0.125 0.0625 0.031 0.015 0.0078 0 |
|  | CAZ | 500 | 0.31 0.15 0.078 0.039 0.019 0.009 0.0048 0 |
| Del68-169_EL | MEM | 500 | 0.0078 0.0039 0.0019 0.0009 0.00048 0.00024 0.00012 0 |
|  | CAZ | 500 | 162 81 40.5 20.25 10.125 5.0625 2.53125 0 |

Abbreviation: CAZ, ceftazidime; MEM, meropenem. -, no data.

**Table S4.** KPC amino acid mutations after resistance development to CZA and CZA-MEM.

| **Strains** | **Type** | **CZA only** | **CZA-MEM** |
| --- | --- | --- | --- |
| P05 | KPC-2 | **L169P**, T243A, H273R | No mutation |
| P13 | KPC-2 | M50I, P175Q | **D179Y** |
| P16 | KPC-2 | T243P | - |
| P18 | KPC-2 | **P174L** | - |
| P27 | KPC-2 | Ins241_E | - |
| P31 | KPC-2 | **L169P**, T243A, H273R | - |
| P36 | KPC-2 | NA | - |
| P39 | KPC-2 | **Ins171_EV** | - |
| P51 | KPC-2 | Y241D | - |
| P56 | KPC-2 | Ins260_AVYTRAPNKDDKHSE | - |
| P60 | KPC-2 | **P174L** | **Del168-169_EL** |
| P77 | KPC-2 | **D179N** | - |
| P85 | KPC-2 | **Del165-167_WEL**, Ins164_R | - |
| H10 | KPC-2 | **D176N** | **R164L/ T243S** |
| H29 | KPC-2 | **P174L** | **R164S/ Ins275_NKDD** |
| H66 | KPC-2 | P67L | - |
| H69 | KPC-2 | T243P | - |
| H80 | KPC-2 | **L169P**, T243A, H273R | - |
| KP13 | KPC-2 | NA | - |
| K15 | KPC-2 | **L169P** | - |
| 10F81 | KPC-3 | **L169P**, T243G | - |
| 09C44 | KPC-3 | **Del168-169_EL**,  Ins268_ VLAVYTRAPN, Y241S | - |
| 09D16 | KPC-3 | **P174L**, T243G | - |
| 10E34 | KPC-3 | **P174L**, T243G | - |
| 1-36 | KPC-3 | D179A | - |
| 11F13 | KPC-3 | **P174L** | - |

Del, deletion; Ins, insertion. NA, the strain was not saved or was lost after preservation.

Blank (-), the corresponding strain could not be induced to evolve at the corresponding concentration of CZA-MEM. Bold letters indicate that the amino acid mutation site is within the Ω loop. Strain K15 was of animal origin.

**Table S5.** β-lactamase mutations in 5 *Klebsiella pneumoniae* strains.

| Strains | KPC-2 | CTX | TEM | OXA-48 | SHV |
| --- | --- | --- | --- | --- | --- |
| P05 | No mutations | No mutations | No mutations | / | No mutations |
| P13 | D179Y | No mutations | No mutations | No mutations | / |
| P60 | Del168-169_EL | No mutations | No mutations | No mutations | No mutations |
| H10 | R164L/T243S | / | / | No mutations | / |
| H29 | R164S/Ins275_NKDD | / | / | / | / |

Del, deletion; Ins, insertion.

Blank (/), the strain does not have the corresponding resistance gene.

**Figure S1.** Characteristics of the 26 KPC-Kp isolates used in this study. The heatmap shows antimicrobial susceptibility data (MIC values) together with genotypic features, including KPC type, sequence type (ST), resistance genes, plasmid replicon types, and virulence genes. All genotypic data were identified by whole-genome sequencing. Antibiotic abbreviations: CZA, ceftazidime-avibactam; CAZ, ceftazidime; MEM, meropenem; PMB, polymyxin B; CS, colistin; AMK, amikacin; GEN, gentamicin; FOS, fosfomycin; TGC, tigecycline; AP, apramycin.

**Figure S2. Gel electrophoresis of pBAD24-*bla*_KPC_ positive clones.** M, 2000 marker; 1, the pBAD24 in DH5α; 2, the pBAD24-*bla*_KPC-2_ in DH5α; 3, pBAD24-*bla*_KPC-2-R164L_ in DH5α; 4, the pBAD24-*bla*_KPC-2-R164L/T243S_ in DH5α; 5, the pBAD24-*bla*_KPC-2-T243S_ in DH5α; 6, the pBAD24-*bla*_KPC-2-R164S_ in DH5α; 7, pBAD24-*bla*_KPC-2-R164S/Ins275_NKDD_ in DH5α; 8, pBAD24-*bla*_KPC-2-D179Y_ in DH5α; 9, the pBAD24-*bla*_KPC-2-Del168-169_EL_ in DH5α; 10, negative control.

**Figure S3. Gel electrophoresis of pET28a-*bla*_KPC_ positive clones.** M, 2000 marker; 1, the pET28a in BL21 (DE3); 2, the pET28a-*bla*_KPC-2_ in BL21 (DE3); 3, pET28a-*bla*_KPC-2-R164L_ in BL21 (DE3); 4, the pET28a-*bla*_KPC-2-R164L/T243S_ in BL21 (DE3); 5, the pET28a-*bla*_KPC-2-T243S_ in BL21 (DE3); 6, the pET28a-*bla*_KPC-2-R164S_ in BL21 (DE3); 7, pET28a-*bla*_KPC-2-R164S_/_Ins275_NKDD_ in BL21 (DE3); 8, pET28a-*bla*_KPC-2-D179Y_ in BL21 (DE3); 9, the pET28a-*bla*_KPC-2-Del168-169_EL_ in BL21 (DE3); 10, negative control.

**Figure S4. SDS-polyacrylamide gel electrophoresis of purified KPC and variants.** M, 180 KDa Protein Marker; 1, KPC-2; 2, KPC-2-R164L; 3, KPC-2-R164L/T243S; 4, KPC-2-T243S; 5, KPC-2-R164S; 6, KPC-2-R164S/Ins275_NKDD; 7, KPC-2-D179Y; 8, KPC-2-Del168-169_EL; 9, negative control.

**References:**

1.	Tsivkovski, R., and O. Lomovskaya**.** 2020. Potency of vaborbactam is less affected than that of avibactam in strains producing KPC-2 mutations that confer resistance to ceftazidime-avibactam. *Antimicrob Agents* *Chemother* 64**:**e01936-19.
